# Internet psychotherapeutic interventions for anxiety disorders – a critical evaluation

**DOI:** 10.1186/s12888-022-04002-1

**Published:** 2022-06-29

**Authors:** Borwin Bandelow, Dirk Wedekind

**Affiliations:** Department of Psychiatry and Psychotherapy, University Medical Centre, Göttingen (UMG), D-37075 Göttingen, Germany

**Keywords:** Anxiety disorders, Panic disorder, Generalised anxiety disorder, Social anxiety disorder, Internet psychotherapy, Meta-analysis

## Abstract

**Background:**

During the COVID-19 pandemic, internet-delivered psychotherapeutic interventions (IPI) move increasingly into the focus of attention.

**Method:**

We reviewed 39 randomized controlled studies of IPIs with 97 study arms (*n* = 4122 patients) for anxiety disorders (panic disorder/agoraphobia, generalized anxiety disorder, and social anxiety disorder) and performed a meta-analysis. Most studies were conducted with cognitive behavioural approaches (iCBT). Results were compared with a previous meta-analysis examining medications and face-to-face (F2F) psychotherapy.

**Results:**

In direct comparisons, IPIs were as effective as F2F-CBT and superior to waitlist controls. Programs with more intensive therapist contact yielded higher effect sizes (ES).

We compared the obtained ES with a previous comprehensive meta-analysis of 234 studies. In this comparison, iCBT was less effective than individual F2F-CBT and medications, not different from pill placebos, and more effective than psychological placebo and waitlist (*p* > .0001 for all comparisons).

ES of IPIs may be overestimated. Treatments were only compared to waitlist, which is not a sufficient control condition. 97% of the studies were not blinded with regard to the main outcome measure. 32% of the participants received antianxiety drugs during the trials. In 89%, participants were recruited by advertisements rather than from clinical settings, and 63% of the participants had an academic background (students or university employees) which might affect the generalizability of the findings.

Remote diagnoses were often made by students without completed training in psychotherapy. In only 15% of the studies, diagnoses were made in personal contact with a psychiatrist or psychologist. In 44% of the studies, the ‘therapists’ maintaining remote contact with the participants were mostly students without completed psychotherapy education.

**Conclusions:**

IPIs may be a useful tool when face-to-face psychotherapy is not easily available, or as an add-on to standard psychotherapeutic or psychopharmacological treatments but should perhaps not be used as monotherapy. We have suggested standards for future research and the practical use of IPIs.

**Supplementary Information:**

The online version contains supplementary material available at 10.1186/s12888-022-04002-1.

## Introduction

During the COVID-19 pandemic, internet-delivered psychotherapy interventions (IPI) for mental disorders move increasingly into focus of attention because personal contacts can be avoided with these interventions. Moreover, IPIs are less expensive, save therapist time, require less organizational efforts and can be used at any time of the day. Anxiety disorders are the most prevalent mental disorders [1] and 26% of all psychotherapies are done with patients with anxiety disorders [2]. As many individuals with anxiety disorders, in particular with social phobia, are reluctant to utilize mental health services because of the stigma associated with mental disorders, availability of IPIs may increase the treatment rates in this population.

The aim of this meta-analysis was to take a critical look at IPIs for anxiety disorders and to determine whether these treatment modalities can serve as a full replacement for standard treatments.

Randomized controlled studies (RCTs) with IPIs mostly recruit their participants via newspaper advertisements or websites. In order to be eligible, participants have to complete diagnostic tests on the website. Some programs include structured diagnostic interviews administered over the telephone or a video link. Usually, participants have to work through computerized modules specially developed for anxiety disorders, which are based on standard psychotherapeutic techniques (mostly cognitive behavioral therapy-oriented approaches), and can be modified to accommodate the special requirements of individual participants. The modules require about as much patient time as face-to-face (F2F) psychotherapy and often involve “homework”, e.g. self-exposure to threatening stimuli in phobic disorders. In most studies, participants can contact therapists via E-mail. However, the time therapists spend communicating with the patients is usually limited because saving therapist time is one of the goals of IPIs. After treatment termination, patients usually rate their improvement by using self-rating scales on the website.

Conducting an RCT on a website is much easier than doing a study with personal contact, as not only the therapeutic contact with participants is much shorter but also the time spent for recruiting patients, completing forms and diagnostic tests. The forms are completed by the participants on the website and can go directly to the electronic database, which can automatically perform the statistical evaluation. This substantial time-saving effect may explain why so many clinical studies on IPIs have been published in the recent years.

Earlier meta-analyses of IPIs for anxiety disorders, e.g. [3], have shown superiority over waitlist control conditions.

In a previous meta-analysis on treatments for anxiety disorders [4], we did not only look at the effect size (ES) differences between the active treatment and the control group (treated vs. control ES) but also determined pre-post ES. The use of pre-post effect sizes has been critisised [5]. One reason is that baseline and post-test scores are not independent of each other, while the correlation of both scores is almost never reported in clinical studies. Moreover, pre-post ES do not only measure the true effect of a treatment but also comprise unspecific effects like spontaneous remission or tendency of regression to the mean. However, when patient populations are the same in the studies being compared, it can be assumed that the correlations of pre and post scores and the unspecific effects are similar. On the other hand, pre-post ES have the advantage that they can be used to determine the absolute improvement of treatments. When studies are compared that have different control conditions, measuring pre-post ES is the only way to obtain a fair comparison. For example, drugs are mostly compared to a placebo, which has an average pre-post ES of Cohen’s d = 1.3, while waitlist control conditions have a much lower average pre-post ES of d = 0.2. The difference between an average drug and a pill placebo was around 2.0–1.3 = 0.7 (treated vs. control ES), according to our analysis, while the difference between a psychological therapy and a waitlist was 1.3–0.2 = 1.1. Thus, on the basis of treated vs. control ES, psychological therapies would seem to be the more effective treatment. However, in absolute terms, the average ES of psychotherapies is much less than the one of medications. Patients are only interested in a large absolute decrease of their symptoms from pre to post, not in the relative difference to a control group. Therefore, when trials using different control groups are being compared, using pre-post ES is the adequate method [4, 6, 7]. Moreover, the pre-post method allows to include not only the few direct comparisons, but many more studies, e.g. those RCTs that do not have an inactive control group, for instance when different techniques are compared.

We compared the ES from the present meta-analysis with the data obtained in our previous analysis which examined F2F psychotherapy and medications for anxiety disorders.

## Methods

We included randomized controlled trials (RCTs) comparing IPIs with an inactive (e.g. waitlist) or active (e.g. F2F-CBT) control group in patients aged 18 or over who met criteria for PDA, GAD, and SAD according to DSM-IV or later versions. Abstracts were identified by searching for the terms “internet”, “randomized”, and “treatment” using MEDLINE, ISI Web of Science, and hand search (PRISMA statement [8], Supplemental Appendix: supplementary Fig. 1; supplementary table 1) through December 2020. We also used a large database of all studies for anxiety disorders collected for the German guidelines for anxiety disorders [9] which was updated for the 2021 version of the guideline [10].

After screening by title and abstract, full texts were assessed for eligibility. Quality of study reporting was assessed by using the CONSORT 2010 Statement [11]. Reasons for exclusion were: Reviews/comments, open studies, case reports, samples including patients with no primary anxiety disorder diagnosis or with mixed diagnoses (e.g. including participants with more than one anxiety disorder or other mental disorders), samples containing only subgroups (e. g. only high school students), secondary analyses, studies not fulfilling inclusion criteria (e. g. studies not comparing comparing IPIs with an inactive or active control), studies only reporting follow-up analyses of a previous study, combination treatments, missing information making it impossible to compute ES, or an evaluable sample size of any of the treatment arms < 10. We extracted the following data from the included studies: participant characteristics (baseline symptom levels, mean age, gender, referral type, and educational level), support during intervention, adherence to the study protocol, which was defined as the percentage of randomized participants who finished the course, diagnostic scales, information about sequence generation, allocation concealment, blinding, and drop-out analysis.

We compared the results of the pre-post ES of this meta-analysis with a previous meta-analysis done with the same method [4] which included all evaluable RCTs with F2F psychotherapies (*n* = 94; 6922 patients) and common antianxiety medications, e.g. antidepressants (*n* = 110; 28,051 patients). Inclusion and exclusion criteria and assessments of study quality were similar in both studies so that the study samples were comparable.

### Meta-analytical procedure

Two reviewers (BB and DW) independently extracted all data, with differences resolved following discussion. Interrater reliability for decisions about whether to include or exclude a study was κ = 0.88. In order to limit heterogeneity and to achieve maximum comparability, we preferably used the most commonly applied scales, i.e. the Panic Disorder Severity Scale (PDSS) for PDA, the Penn State Worry Questionnaire (PSWQ) and GAD and the Liebowitz Social Anxiety Scale (LSAS) for SAD. These were not necessarily defined as primary efficacy measures in the studies. If these scales were not available, we used other specific symptoms scales following a hierarchy [4]. All scales were self-rated by the participants.

All inactive control conditions used in the RCTs were waitlist controls. All active controls were F2F-CBT conditions.

Statistical analysis was done using Comprehensive Meta-Analysis 3. ES (Cohen’s d) were calculated as (1) treated vs. control ES (post-test difference between the mean of the treatment condition and the mean of the control condition), or (2) pre-post ES, in both cases divided by the pooled pre-treatment standard deviation and adjusted for sample size. We used Cohen’s d instead of Hedges’ g because the sample sizes were relative large (> 20 in all treatment arms). Because of the assumption of heterogeneous populations in the different studies, and because moderate (I^2^ > 50%) to high (> 75%) heterogeneity was found for most comparisons, the random-effects model was used in all analyses. To assess publication biases, p values for Egger’s regression intercept (a method to quantify the bias captured by a funnel plot) for between group ES sizes, and ES adjusted for publication bias using Duval and Tweedie’s ‘‘trim and fill’’ method, which is used to correct the funnel plot by estimating the number of missing studies and the ES of these studies, were determined.

We preferably used intent-to-treat (ITT) data based on the last observation carried forward (LOCF) method for all studies, which were reported in all but 2 studies. The ES for the 3 anxiety disorders were pooled in order to improve the statistical validity and to reduce the influence of heterogeneity by increasing the number of studies and to avoid multiple testing, which would have arisen if the 3 disorders were tested separately. Nevertheless, we also determined the pre-post ES of the 3 disorders separately.

The search identified 7 studies which directly compared iCBT with F2F-CBT. We also contrasted the ES in 8 studies in which treatment arms with minimal or no therapist support were compared to more intensive therapist support (e.g. up to three 15-min weekly contacts per E-mail).

The statistical methods of the comparison of the two meta-analyses are shown in the Supplemental Appendix.

## Results

We identified 39 studies (PDA, *n* = 11; GAD, *n* = 8; SAD, *n* = 20) with 97 treatment arms. IPI arms were mostly based on CBT approaches (iCBT; *n* = 57); 2 were done with Psychodynamic Therapy (iPDTh) and Applied Relaxation (iAR), and 1 with Interpersonal Therapy (iIPT).

### Treated vs. control ES

In the 31 studies in which IPIs were directly compared to a waitlist condition (treated vs. control ES), a significant difference to waitlist was found for iCBT, iPDTh, and iAR (Table 1; see also Supplemental Appendix: Forest plot: supplementary Fig. 2; funnel plots: supplementary Figs. 3 and 4). Running the analysis without one extreme outlier [12] did not change the overall result. In 7 studies comparing iCBT with F2F-CBT, no significant difference was found (Table 1; see also Supplemental Appendix: Forrest plot: supplementary Fig. 5; funnel plot: supplementary Fig. 6). In the 10 studies comparing minimal or no therapist support with more intensive therapist support, high intensity contact was significantly more effective; however, the mean difference was marginal (Table 1; see also Supplemental Appendix: Forrest plot: supplementary Fig. 7; funnel plots: supplementary Fig. 8).

**Table 1:** Treated vs. control effect sizes: IPIs vs. waitlist, iCBT vs. F2F-CBT; high vs. low intensity contact

**Treatment**	*n arms*	*d*	*CI*	*p*	*I*^*2*^	*Publication Bias*		
						*Egger p*	*adjusted d*	*CI*
IPIs vs. waitlist	31	1.08*	0.91–1.26	> .0001	71.7	.0007	1.08	0.91–1.26
iCBT	28	1.12	0.93–1.30	> .0001	73.1	.0006	1.11	1.93–1.30
iPTh	2	0.67	0.10–1.24	.021	0	–	–	–
iAR	1	1.03	0.52–1.54	> .0001	–	–	–	–
iCBT vs. F2F-CBT	7	0.10	-0.16–0.35	0.45 (N.S.)	44.6	.29 (N.S.)	0.10	-0.16–0.35
High vs. low intensity contact	8	0.13	0.004–0.26	.04	0	.26 (N.S.)	0.09	0.06–0.25

*iCBT* Internet-delivered Cognitive Behavioural Therapy, *F2F-CBT* Face-to-face Cognitive Behavioural Therapy, *iPDTh* Internet-delivered Psychodynamic Therapy, *iAR* Internet-delivered Applied Relaxation, *iIPT* Internet-delivered Interpersonal Therapy

*n* Number of studies, *d* Effect size Cohen’s d, *CI* Confidence interval, *I*^*2*^ Heterogeneity, *Egger p* *P* values for Egger’s regression intercept, *adjusted d* Adjusted Cohen’s d after applying Duval and Tweedie’s trim and fill method

^*^After excluding one extreme outlier, d was 1.03* (CI 0.88–1.19); *p* > .0001

### Pre-post ES and comparison with previous meta-analysis

Pre-post ES of the treatment arms are tabulated in Table 2.

**Table 2:** Pre-post ES of interventions (study arms). Random effects model. Abbreviations see Table 1

**Treatment**	*n arms*	*n patients*	*d*	*CI*	*I*^*2*^
All IPIs	62	3244	1.20	1.10–1.30	64.7
iCBT	57	2844	1.21	1.10–1.31	66.3
PDA	19	694	1.30	1.03–1.58	74.8
GAD	11	870	1.28	1.11–1.45	56.0
SAD	27	1280	1.15	1.00–1.29	61.2
F2F-CBT	7	215	1.28	0.83–1.73	79.1
iPDTh	2	61	1.35	0.96–1.73	0
iAR	1	40	1.02	0.55–1.49	0
iIPT	1	19	0.49	-0.12–1.10	-
Waitlist	23	720	0.20	0.09–0.31	13.9

**Fig. 1:**
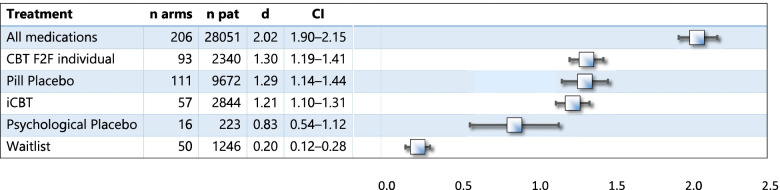
Pre-post ES of iCBT in comparison to previous meta-analysis [4]

We compared the findings for iCBT with our previous meta-analysis [4] by using pre-post ES (Fig. 1, Table 3). In this comparison with a large database, iCBT was significantly less effective than anxiety medications and F2F-CBT, not different from pill placebo, and more effective than psychological placebo and wait list.

**Table 3:** Comparison with meta-analysis [4]. All tests remained significant after Bonferroni correction. p, difference to iCBT. Other abbreviations see Table 1

**Treatment**	*n arms*	*n patients*	*d*	*CI*	p*
iCBT	57	2844	1.21	1.10–1.31	
All medications	206	28,051	2.02	1.90–2.15	> .0001^a^
CBT F2F individual	93	2340	1.30	1.19–1.41	> .0001^a^
Pill Placebo	111	9672	1.29	1.14–1.44	0.18 (N.S.)
Psychological Placebo	16	223	0.83	0.54–1.12	> .0001^b^
Waitlist	50	1246	0.20	0.12–0.28	> .0001^b^

^*^all *p* values significant after Bonferroni correction

^a^more effective than iCBT

^b^less effective than iCBT

### Characteristics of IPI studies

The study characteristics are listed in Table 4. The mean duration of studies was 9.3 weeks. Average therapist time was 17.5 min per week. The average adherence in the studies was 69.3%.

**Table 4:** Study characteristics (therapists, participants). Weighted means

Diagnoses	
Panic disorder	*N* = 11 (28.2%)
Generalised anxiety disorder	*N* = 8 (20.5%)
Social anxiety disorder	*N* = 20 (51.3%)
Total	*N* = 39
Study arms	*N* = 97
Average sample size per study arm	*N* = 42.5 (SD 28.3)
Referral	
newspaper advertisements/websites	89.7%
general practitioners, psychiatrists or psychologists	10.3%
Diagnosis made by	
only psychologists/psychiatrists	33.3%
students and psychologists	41.0%
self diagnosis via website	5.1%
trained interviewers	2.6%
no information	12.8%
Diagnosis made	
in person	25.6%
telephone	64.1%
self diagnosis via website	5.1%
no information	5.1%
Diagnoses made by personal contact with psychiatrist/psychologist	15.4%
Mean duration of studies (weeks)	9.3 (SD 1.9)
Therapist time (minutes per week)	17.5
Therapists	
Clinicians	51.3%
Mostly students	41.0%
None at all	7.7%
Participants with academic background	63.0%
Blinding of main outcome	2.6%
Ongoing psychopharmacological medications allowed	
Yes	87.1%
No	2.6%
No information	10.3%
Average percentage of patients receiving ongoing medication	31.3%
Adherence	69.3%
Average age of participants	
Panic disorder/agoraphobia	38.6 years
Generalised anxiety disorder	38.2 years
Social anxiety disorder	35.5 years

#### Limitations of the studies

In the majority of studies (88.7%), patients were not referred from clinical settings but were recruited by newspaper advertisements or websites. With the exception of one study, the main outcomes of the studies were not blinded, as they were based on self-report questionnaires and the patients knew that they were in the active treatment condition. Most studies (87.1%) allowed concomitant psychopharmacological drugs when these treatments had not been changed in the last 4–12 weeks, and 31.3% of the patients stated that they were taking medication during the trial. In the 22 studies giving details on education level of participants, a high percentage had an academic background (63.0%).

We compared the age of the participants in the studies with a database on the average age of participants in 832 clinical studies [13]. The mean age (PDA: 38.6, GAD 38.2; SAD 35.7) in the evaluated studies was similar to the typical age of participants calculated in this database (PDA: 37.2, GAD 40.7; SAD 35.2).

In order to find out whether the participants recruited for the IPI studies were less severely ill than those included in average clinical studies, we compared the baseline severity scores with the ones obtained in our first meta-analysis. For SAD, the weighted average baseline score of the Liebowitz Anxiety Scale (LSAS) of the IPIs was 72.7 ± 21.9, which was significantly lower than the baseline scores in average drug studies (84.0 ± 8.3; df = 79; t = 50.2; *p* < 0.0001). There was only a trend for lower severity in comparison to F2F psychotherapy studies (73.2 ± 11.0; t = 1.5, *p* = 0.07). For PDA and GAD, we did not have sufficient data to compare the baseline values with the same scale.

In only one of the studies it was mentioned that the participants were provided with an emergency telephone number in case of emerging serious mental problems, e.g. suicidal ideas.

#### Sample sizes

Average sample sizes of IPIs are significantly larger (41.5 ± 28.3 per study arm; *n* = 97) than in the F2F studies in our previous meta-analysis (29.7 ± 25.5; *n* = 175; *p* < 0.0001).

#### Study quality

The number of 38 CONSORT quality indicators not fulfilled ranged from 2 to 13, with an average of 4.4 ± 2.1 per study. The most frequent violations of CONSORT items were: no adequate blinding (97.4%), no report of adverse effects or harms (92.3%), missing power calculation or sample size too small for non-inferiority trial (59.0%), lack of generalizability (53.8%), no a priori trial registration 46.2%, randomization method not described adequately (20.5%), no primary efficacy measure defined (10.3%), funding not reported (10.3%), and no intent-to-treat evaluation (5.1%).

#### Allegiance effects

As all studies were conducted by work groups that support the use of IPIs, we could not perform a comparison of the effect sizes of studies conducted by “supporters” vs. studies from independent work groups.

#### Publication bias

There was evidence for publication bias in studies comparing IPI and iCBT vs. waitlist in the sense of “small study effects”. However, this would lead only to a minimal adjustment according to Duval and Tweedie’s trim and fill method of the ES difference of iCBT vs. waitlist from d = 1.12 to 1.11.

## Discussion

During the Covid-19 pandemic, psychological therapies delivered via internet (IPI) may become increasingly more important, as personal contact or travel can be avoided. Moreover, IPIs have the advantage to be much less expensive, can be done at any time of the day and are not dependent of local availability of therapists.

In the present meta-analysis, IPIs were significantly superior to waitlist controls. However, a waitlist control is an easy target to beat. Even treatments that are significantly less effective than a pill placebo or a psychological placebo would pass this test, as waitlist conditions yield only very small average ES of around d = 0.2 [4]. They have been shown to be even less effective than a no-treatment condition [14], as those participants allocated to waitlist may be motivated to overstress their illness symptoms at the end of the waiting period, for having a justification that they can receive their originally desired therapy. Moreover, patients may be demoralized during the waiting period because they have consented not to start any other treatments. Being allocated to a waitlist could be therefore regarded a “nocebo” condition. Moreover, when a psychological treatment is superior to a waitlist control, this only shows that there is some unspecific effect but does not provide information whether the treatment has specific psychotherapy effects characteristic for a certain technique. Therefore, the scientific value of waitlist-controlled RCTs is limited.

The more important question is how IPIs perform relative to F2F-CBT and other alternative treatments. In the 7 available direct comparisons of iCBT and F2F-CBT, no significant difference was found. The results may be less reliable due to the low number of direct comparisons. Moreover, as all these 7 comparisons were done by research teams promoting iCBT, these results may be biased by allegiance effects. In contrast to an earlier meta-analysis which also did not find a difference between IPI and F2F-CBT [15], we were able to compare iCBT with a much larger number of F2F-CBT studies (*n* = 93) by using the pre-post ES, and found a significant difference in favor of personal interventions. However, this difference of d = 0.09 was so small that it might hardly be noticeable by the patient. As findings in psychotherapy research suggest that unspecific therapist effects in psychotherapy, like life experience, empathy, warmth, positive regard, therapeutic alliance, transference, or clinical experience have an important influence on the outcome of treatment [16], a much larger difference should have been expected. In meta-analyses, small but significant correlations have been found between patients’ expectations perception of treatment credibility, therapeutic alliance and outcome alliance [17–19]. Also, therapists with a degree in psychology or medicine, many years of psychotherapeutic education and clinical experience should perform much better than a computer program accompanied by short E-mails from psychology students. Notably, the treatment duration in the IPIs was only 9.3 weeks on average, while F2F psychotherapy studies have a mean duration of 12.4 weeks, according to our first meta-analysis.

However, these results have to be interpreted with caution because there are reasons to assume that the efficacy of IPIs may have been overestimated in the evaluated studies.

- With the exception of one study, the main outcomes of the studies were not blinded, as they were based on self-report questionnaires and the patients knew that they were in the active treatment condition. It was reported that ES were exaggerated by 68% in unblinded psychotherapy studies [20].
- Most of the studies allowed ongoing psychopharmacological treatments, and around 30% of the participants were on continuing drug treatment during the study. Therefore, the calculated pre-post ES were actually a sum of psychological and psychopharmacological effects in a substantial number of patients. However, this also applies to average studies conducted with F2F psychotherapy.
- The IPI studies included patients who may be more prone to respond than average anxiety patients, as most of the study participants had been recruited from nonclinical settings, two thirds had an academic background, and familiarity with the use of computers is required for this special treatment modality. A meta-analysis found that in open recruitment studies in which participants referred themselves, the ESs were higher than in studies in which patients were recruited from clinical settings [15]. We found that the baseline scale scores in SAD patients in the IPIs were significantly lower than the scores in average medication studies but did not differ from the average baseline scores in F2F psychotherapy studies.
- Allegiance effects cannot be excluded in all included IPI studies. In our previous meta-analysis, we found possible allegiance effects in 32.6% of the F2F psychotherapy studies.

In the few studies comparing high and low intensity of therapist contact it was found that the more patient-therapist interaction (e.g. via E-mail or telephone) was involved, the higher were the ES.

If participants applying for IPIs were substantially younger than average patients in F2F studies, this would affect the generalizability of the findings. However, we found that the mean age of treated subjects was similar to average clinical studies with these disorders.

Average sample sizes of IPIs are significantly larger than in typical F2F studies, perhaps because recruiting participants for IPIs is less complex and expensive than for F2F studies. Thus, the reliability of the results is larger than in average psychotherapy studies.

The average adherence in the studies was 69.3%, which is in the normal range of most clinical studies. As a rule of thumb, one third of the participants drop out in any study of psychiatric or psychological treatments. However, in a systematic review of IPIs, the percentage of participants adhering to the programs varied widely between 7.8–75.0% [21]. In routine application of IPIs, attrition rates may be even higher. In an observational study of a freely available Web-based intervention, an extremely high attrition rate was found, with only 1% out of 1161 of registered users completing the 12-week program [22].

### Limitations

We did not look at the results of follow-up assessments because we had found in a meta-analysis that long-lasting treatment effects observed after the original treatment was stopped may be superimposed by effects of spontaneous remission or regression to the mean and are therefore unreliable [7]. In RCTs involving only a waitlist control, a control group is lacking in the follow-up period.

The results with IPI studies using Psychodynamic Therapy, Applied Relaxation (AR) and Interpersonal Therapy are only preliminary because they are only based on 1 to 2 studies which included very few participants.

AR is seen as a legitimate psychotherapy by some authors (e.g. [23], while it was considered a less effective control condition in RCTs (e.g. [24]) or meta-analyses [25, 26]. It is often used as component in CBT programs. A meta-analysis contrasting AR with CBT without AR did not find a difference in efficacy [27].

A limitation is that the meta-analysis was not preregistered, as according to current quality standards preregistrations are recommended [28].

While the screening and evaluation of the studies was done by two independent raters, the electronic search was only done by on investigator. However, according to recommended standards, [28], also the search should be done independently.

### Proposal for standards

IPIs may be a useful tool when personal psychotherapy is not easily available, or as an add-on to standard psychotherapeutic or psychopharmacological treatments. On average, therapist time can be reduced to approximately one quarter of what is needed for F2F psychotherapy.

Based on our review, we have formulated a proposal for the standards for psychological interventions delivered via internet (Table 5).

**Table 5:** Internet-delivered psychotherapeutic interventions for anxiety disorders: proposal for standards

- Diagnoses should be made by psychiatrists or psychologists in personal contact
- Contact persons on the “other end” should have a degree in medicine or psychology and should have a completed (or almost-completed) training in psychotherapy
- Therapist contact with participants via E-mail, telephone or videoconferencing should be at least 15 min per week
- Program modules should be based on scientific findings on the effective ingredients of psychotherapy and should be developed by experienced psychotherapists
- Patients should be informed about alternative treatments that might yield higher ES, e.g. face-to-face CBT or medications
- E-mail and videoconferencing services must be encrypted
- Participants should be provided with a 24-h emergency telephone number in case of severe mental problems, e.g. suicidal ideas

For future studies on IPIs, we propose:

- Studies should be conducted with representative samples, e.g. they should not be restricted to populations with a preponderance of participants with academic background.
- In order to examine the true effects of IPIs, studies should be conducted with medication-free patients.
- Study assessments should be done by raters blind to treatment allocation in order to minimize expectation effects.
- IPS should not only be compared to waitlist controls but also to psychological placebos. Our meta-analyses showed that IPIs do differ from psychological placebos in the indirect comparison. However, psychological placebos that can be applied as internet application have yet to be developed.

## Conclusion

When compared to face-to-face psychotherapy IPIs showed comparable ES. However, there are some reasons to assume that the ES of IPIs in the available studies are overestimated.

IPIs may be a useful tool when face-to-face psychotherapy is not easily available, or as an add-on to standard treatments but should perhaps not be used as monotherapy. We have proposed standards for future research and the practical use of IPIs.

## Supplementary Information

**Additional file 1.**

## Data Availability

All data generated or analyzed during this study are included in this published article and the Supplemental Appendix.
