# Supplementary material for: Internet psychotherapeutic interventions for anxiety disorders – a critical evaluation
Source: BMC Psychiatry. 2022 Jun 29;22:441. doi: 10.1186/s12888-022-04002-1 (PMC9241282; doi:10.1186/s12888-022-04002-1)
Supplement: Supplementary file 1 — Additional file 1. [file 12888_2022_4002_MOESM1_ESM.docx]

# Supplementary Information

Supplementary table 1. List of included RCTs of IPIs. Abbreviations see Table 1 (main document).

| **Authors** | **Disorder** | **Treatment 1** | **Treatment 2** | **Treatment 3** | **Treatment 4** | **Control** | **Scale** | **total n** | **Duration**  **weeks** |
| --- | --- | --- | --- | --- | --- | --- | --- | --- | --- |
| 1. Allen et al 2016 | PDA | iCBT |  |  |  | waitlist | PDSS | 63 | 5 |
| 1. Andersson et al 2006 | SAD | iCBT |  |  |  | waitlist | LSAS | 68 | 5 |
| 1. Andersson et al 2012 | GAD | iCBT | iPDTh |  |  |  | PSWQ | 81 | 13 |
| 1. Andrews et al 2011 | SAD | iCBT | F2F CBT |  |  |  | SPS | 37 | 8 |
| 1. Berger et al 2009 | SAD | iCBT |  |  |  | waitlist | LSAS | 52 | 10 |
| 1. Berger et al 2011 | SAD | iCBT | iCBT on demand | iCBT guided |  |  | LSAS | 81 | 10 |
| 1. Bergstrom et al 2010 | PDA | iCBT | F2F CBT group |  |  |  | PDSS | 104 | 10 |
| 1. Botella et al 2010 | SAD | iCBT | F2F CBT |  |  | waitlist | SAD | 127 | 9 |
| 1. Carlbring et al 2003 | PDA | iCBT | iAR |  |  |  | BAI | 22 | ? |
| 1. Carlbring et al 2005 | GAD | iCBT | F2F CBT |  |  |  | BAI | 49 | 10 |
| 1. Carlbring et al 2006 | GAD | iCBT |  |  |  | waitlist | BAI | 60 | 10 |
| 1. Carlbring et al 2007 | SAD | iCBT |  |  |  | waitlist | LSAS | 57 | 9 |
| 1. Ciuca et al 2018 | PDA | iCBT | F2F CBT |  |  | waitlist | PDSS | 111 | 12 |
| 1. Dagöö et al 2014 | SAD | iCBT | iIPT |  |  |  | LSAS | 52 | 9 |
| 1. Dahlin et al 2016 | GAD | iCBT |  |  |  | waitlist | PSWQ | 103 | 9 |
| 1. Dear et al 2015 | GAD | iCBT trans­diagnostic | iCBT disorder-specific | iCBT clinician-guided | iCBT self-guided |  | GAD-7 | 582 | 8 |
| 1. Dear et al 2016 | SAD | iCBT trans­diagnostic | iCBT disorder-specific | iCBT clinician-guided | iCBT self-guided |  | Mini-SPIN | 412 | 8 |
| 1. Fogliati et al 2016 | PDA | iCBT trans­diagnostic | iCBT disorder-specific | iCBT clinician-guided | iCBT self-guided |  | PDSS | 264 | 8 |
| 1. Furmark et al 2009 study 1 | SAD | iCBT | Bibli­otherapy |  |  | waitlist | LSAS | 120 | 9 |
| 1. Furmark et al 2009 study 2 | SAD | iCBT | Bibli­otherapy | i-Applied Relaxation iAR |  |  | LSAS | 115 | 9 |
| 1. Gershkovich et al 2017 | SAD | iCBT self-guided | iCBT therapist-guided |  |  |  | LSAS | 115 | 8 |
| 1. Hedman et al 2011 | SAD | iCBT | F2F CBT |  |  | waitlist | LSAS | 42 | 15 |
| 1. Hirsch et al 2018 | SAD | iCBT | iCBT RNT priming |  |  |  | PSWQ | 66 | 9 |
| 1. Johansson et al 2017 | SAD | iPDTh |  |  |  | waitlist | LSAS | 64 | 10 |
| 1. Kiropoulos et al 2008 | SAD | iCBT | F2F CBT |  |  |  | PDSS | 72 | 12 |
| 1. Klein et al 2006 | PDA | iCBT | Bibliotherapy |  |  | waitlist | PDSS | 83 | 6 |
| 1. Klein et al 2009 | PDA | iCBT high intensity contact | low intensity contact |  |  |  | PDSS | 55 | 8 |
| 1. Oromendia et al 2016 | PDA | iCBT scheduled support | iCBT support on demand |  |  | waitlist | PDSS | 77 | 8 |
| 1. Paxling et al 2011 | GAD | iCBT |  |  |  | waitlist | PSWQ | 82 | 8 |
| 1. Richards et al 2006 | PDA | iCBT | iCBT stress management |  |  | waitlist | PDSS | 96 | 8 |
| 1. Robinson et al 2010 | GAD | iCBT clinician-guided | iCBT technician-guided |  |  | waitlist | PSWQ | 145 | 10 |
| 1. Schulz et al 2016 | SAD | iCBT clinician-guided | iCBT clinician-guided group |  |  |  | SPS | 149 | 12 |
| 1. Titov et al 2008c | SAD | iCBT |  |  |  | waitlist | SPS | 99 | 10 |
| 1. Titov et al 2008b | SAD | iCBT |  |  |  | waitlist | SPS | 81 | 10 |
| 1. Titov et al 2008a | SAD | iCBT clinician-guided | iCBT self-guided |  |  |  | SPS | 94 | 10 |
| 1. Titov et al 2009 | SAD | iCBT |  |  |  | waitlist | PSWQ | 45 | 9 |
| 1. Titov et al 2010 | SAD | iCBT | iCBT motivation. enhancm. |  |  |  | SPS | 108 | 9 |
| 1. Tulbure et al 2015 | SAD | iCBT |  |  |  | waitlist | LSAS | 76 | 9 |
| 1. Wims et al 2010 | PDA | iCBT |  |  |  | waitlist | PDSS | 44 | 8 |

## Statistical evaluation

To compare the results of two meta-analyses, we used Cohen’s d and the confidence intervals. The ES difference was δ = d_2_-d_1_. The standard error of the mean was estimated as SE=maximum error margin – minimum error margin)/3.96. The standard deviation for the difference was calculated as σ$=\sqrt{{SE1}^{2}-{SE2}^{2}}$. Assuming a normal distribution the probability F(x) that difference d_1_ and d_2_ was different from 0 was calculated from δ and σ^2^.

Supplementary figure 1. Search algorithm according to the PRISMA-Statement (Moher et al 2011)


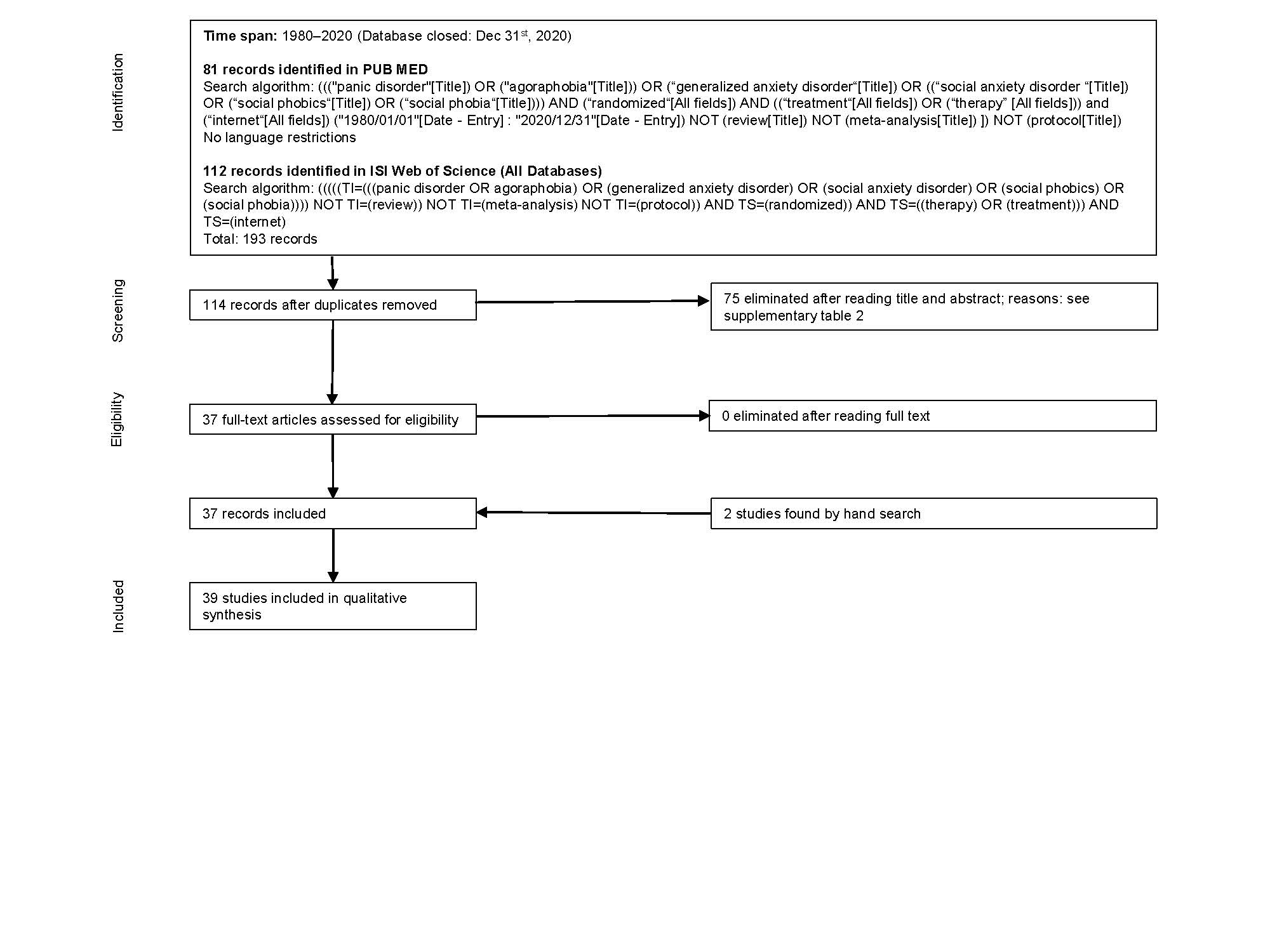


Supplementary figure 2. Forrest Plot: All IPIs vs. Waitlist


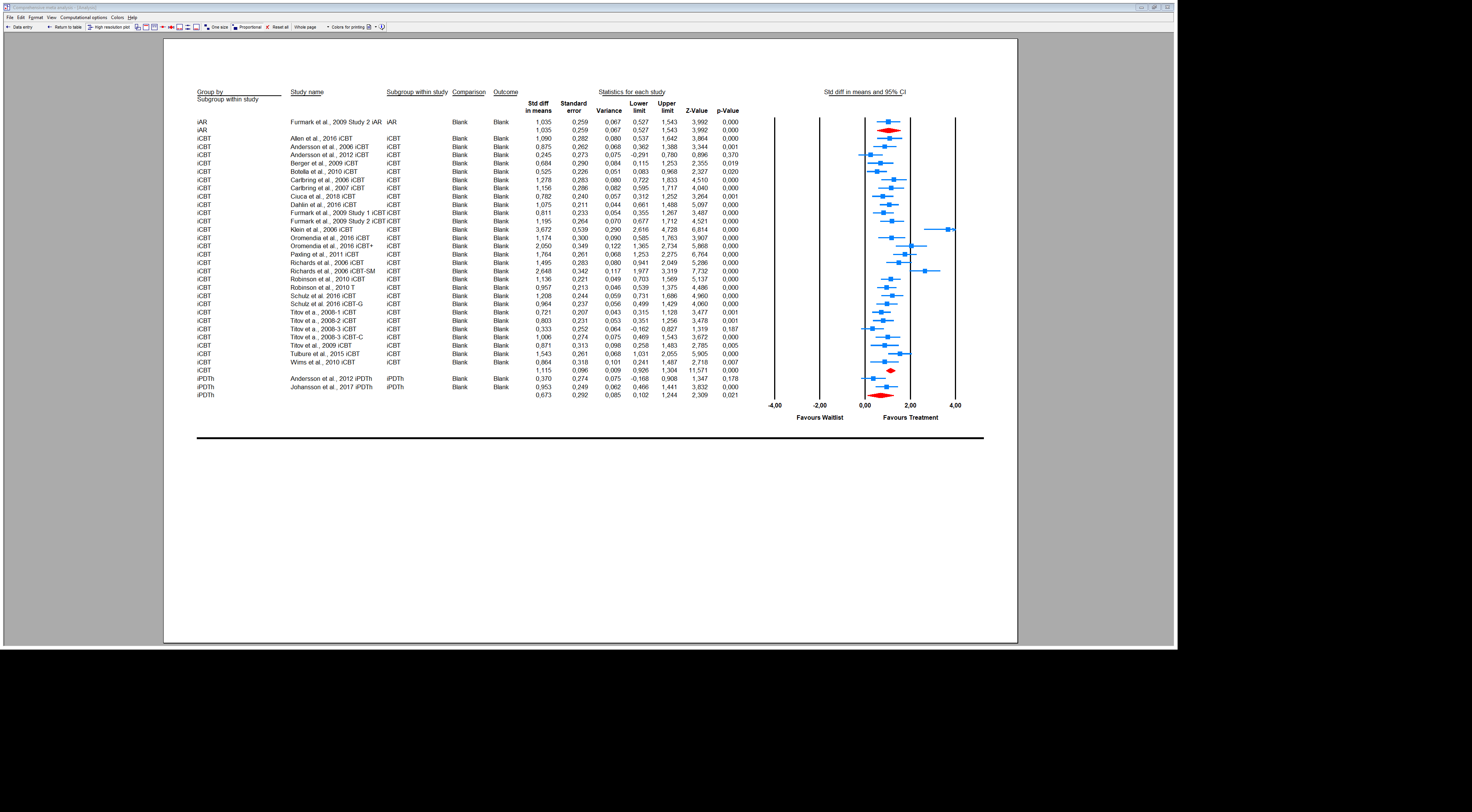


Supplementary figure 3. Funnel Plot: All IPIs vs. Waitlist

Supplementary figure 4. Funnel Plot: iCBT vs Waitlist

Supplementary figure 5. Forrest Plot: iCBT vs. F2F CBT

Supplementary figure 6. Funnel Plot: iCBT vs. F2F-CBT

**Supplementary figure 7. Forrest** **Plot: High vs. low intensity contact**

Supplementary figure 8. Funnel Plot: High vs. low intensity contact

**References**

Allen AR, Newby JM, Mackenzie A, Smith J, Boulton M, Loughnan SA, Andrews G (2016): Internet cognitive-behavioural treatment for panic disorder: randomised controlled trial and evidence of effectiveness in primary care. *BJPsych Open* 2:154-162.

Andersson G, Carlbring P, Holmstrom A, Sparthan E, Furmark T, Nilsson-Ihrfelt E, Buhrman M, Ekselius L (2006): Internet-based self-help with therapist feedback and in vivo group exposure for social phobia: a randomized controlled trial. *J Consult Clin Psychol* 74:677-86.

Andersson G, Paxling B, Roch-Norlund P, Ostman G, Norgren A, Almlov J, Georen L, Breitholtz E, Dahlin M, Cuijpers P, Carlbring P, Silverberg F (2012): Internet-based psychodynamic versus cognitive behavioral guided self-help for generalized anxiety disorder: a randomized controlled trial. *Psychother Psychosom* 81:344-55.

Andrews G, Davies M, Titov N (2011): Effectiveness randomized controlled trial of face to face versus Internet cognitive behaviour therapy for social phobia. *Aust N Z J Psychiatry* 45:337-40.

Berger T, Caspar F, Richardson R, Kneubuhler B, Sutter D, Andersson G (2011): Internet-based treatment of social phobia: a randomized controlled trial comparing unguided with two types of guided self-help. *Behav Res Ther* 49:158-69.

Berger T, Hohl E, Caspar F (2009): Internet-based treatment for social phobia: a randomized controlled trial. *J Clin Psychol* 65:1021-35.

Bergstrom J, Andersson G, Ljotsson B, Ruck C, Andreewitch S, Karlsson A, Carlbring P, Andersson E, Lindefors N (2010): Internet-versus group-administered cognitive behaviour therapy for panic disorder in a psychiatric setting: a randomised trial. *BMC Psychiatry* 10:54.

Botella C, Gallego MJ, Garcia-Palacios A, Guillen V, Banos RM, Quero S, Alcaniz M (2010): An Internet-based self-help treatment for fear of public speaking: a controlled trial. *Cyberpsychol Behav Soc Netw* 13:407-21.

Carlbring P, Bohman S, Brunt S, Buhrman M, Westling BE, Ekselius L, Andersson G (2006): Remote treatment of panic disorder: a randomized trial of internet-based cognitive behavior therapy supplemented with telephone calls. *Am J Psychiatry* 163:2119-25.

Carlbring P, Ekselius L, Andersson G (2003): Treatment of panic disorder via the Internet: a randomized trial of CBT vs. applied relaxation. *J Behav Ther Exp Psychiatry* 34:129-40.

Carlbring P, Gunnarsdottir M, Hedensjo L, Andersson G, Ekselius L, Furmark T (2007): Treatment of social phobia: randomised trial of internet-delivered cognitive-behavioural therapy with telephone support. *Br J Psychiatry* 190:123-8.

Carlbring P, Nilsson-Ihrfelt E, Waara J, Kollenstam C, Buhrman M, Kaldo V, Soderberg M, Ekselius L, Andersson G (2005): Treatment of panic disorder: live therapy vs. self-help via the Internet. *Behav Res Ther* 43:1321-33.

Ciuca AM, Berger T, Crisan LG, Miclea M (2018): Internet-based treatment for panic disorder: A three-arm randomized controlled trial comparing guided (via real-time video sessions) with unguided self-help treatment and a waitlist control. PAXPD study results. *J Anxiety Disord* 56:43-55.

Dagöö J, Asplund RP, Bsenko HA, Hjerling S, Holmberg A, Westh S, Oberg L, Ljotsson B, Carlbring P, Furmark T, Andersson G (2014): Cognitive behavior therapy versus interpersonal psychotherapy for social anxiety disorder delivered via smartphone and computer: a randomized controlled trial. *J Anxiety Disord* 28:410-7.

Dahlin M, Andersson G, Magnusson K, Johansson T, Sjogren J, Hakansson A, Pettersson M, Kadowaki A, Cuijpers P, Carlbring P (2016): Internet-delivered acceptance-based behaviour therapy for generalized anxiety disorder: A randomized controlled trial. *Behav Res Ther* 77:86-95.

Dear BF, Staples LG, Terides MD, Fogliati VJ, Sheehan J, Johnston L, Kayrouz R, Dear R, McEvoy PM, Titov N (2016): Transdiagnostic versus disorder-specific and clinician-guided versus self-guided internet-delivered treatment for Social Anxiety Disorder and comorbid disorders: A randomized controlled trial. *J Anxiety Disord* 42:30-44.

Dear BF, Staples LG, Terides MD, Karin E, Zou J, Johnston L, Gandy M, Fogliati VJ, Wootton BM, McEvoy PM, Titov N (2015): Transdiagnostic versus disorder-specific and clinician-guided versus self-guided internet-delivered treatment for generalized anxiety disorder and comorbid disorders: A randomized controlled trial. *J Anxiety Disord* 36:63-77.

Fogliati VJ, Dear BF, Staples LG, Terides MD, Sheehan J, Johnston L, Kayrouz R, Dear R, McEvoy PM, Titov N (2016): Disorder-specific versus transdiagnostic and clinician-guided versus self-guided internet-delivered treatment for panic disorder and comorbid disorders: A randomized controlled trial. *J Anxiety Disord* 39:88-102.

Furmark T, Carlbring P, Hedman E, et al (2009): Guided and unguided self-help for social anxiety disorder: randomised controlled trial. *Br J Psychiatry* 195:440-7.

Gershkovich M, Herbert JD, Forman EM, Schumacher LM, Fischer LE (2017): Internet-Delivered Acceptance-Based Cognitive-Behavioral Intervention for Social Anxiety Disorder With and Without Therapist Support: A Randomized Trial. *Behav Modif* 41:583-608.

Hedman E, Andersson G, Ljotsson B, Andersson E, Ruck C, Mortberg E, Lindefors N (2011): Internet-based cognitive behavior therapy vs. cognitive behavioral group therapy for social anxiety disorder: a randomized controlled non-inferiority trial. *PLoS One* 6:e18001.

Hirsch CR, Krahe C, Whyte J, Loizou S, Bridge L, Norton S, Mathews A (2018): Interpretation training to target repetitive negative thinking in generalized anxiety disorder and depression. *J Consult Clin Psychol* 86:1017-1030.

Johansson R, Hesslow T, Ljotsson B, Jansson A, Jonsson L, Fardig S, Karlsson J, Hesser H, Frederick RJ, Lilliengren P, Carlbring P, Andersson G (2017): Internet-based affect-focused psychodynamic therapy for social anxiety disorder: A randomized controlled trial with 2-year follow-up. *Psychotherapy (Chic)* 54:351-360.

Kiropoulos LA, Klein B, Austin DW, Gilson K, Pier C, Mitchell J, Ciechomski L (2008): Is internet-based CBT for panic disorder and agoraphobia as effective as face-to-face CBT? *J Anxiety Disord* 22:1273-84.

Klein B, Austin D, Pier C, Kiropoulos L, Shandley K, Mitchell J, Gilson K, Ciechomski L (2009): Internet-based treatment for panic disorder: does frequency of therapist contact make a difference? *Cogn Behav Ther* 38:100-13.

Klein B, Richards JC, Austin DW (2006): Efficacy of internet therapy for panic disorder. *Journal of Behavior Therapy and Experimental Psychiatry* 37:213-238.

Moher D, Altman DG, Liberati A, Tetzlaff J (2011): PRISMA statement. *Epidemiology* 22:128.

Oromendia P, Orrego J, Bonillo A, Molinuevo B (2016): Internet-based self-help treatment for panic disorder: a randomized controlled trial comparing mandatory versus optional complementary psychological support. *Cogn Behav Ther* 45:270-86.

Paxling B, Almlov J, Dahlin M, Carlbring P, Breitholtz E, Eriksson T, Andersson G (2011): Guided internet-delivered cognitive behavior therapy for generalized anxiety disorder: a randomized controlled trial. *Cogn Behav Ther* 40:159-73.

Richards JC, Klein B, Austin DW (2006): Internet cognitive behavioural therapy for panic disorder: Does the inclusion of stress management information improve end-state functioning? *Clinical Psychologist* 10:2-15.

Robinson E, Titov N, Andrews G, McIntyre K, Schwencke G, Solley K (2010): Internet treatment for generalized anxiety disorder: a randomized controlled trial comparing clinician vs. technician assistance. *PLoS One* 5:e10942.

Schulz A, Stolz T, Vincent A, Krieger T, Andersson G, Berger T (2016): A sorrow shared is a sorrow halved? A three-arm randomized controlled trial comparing internet-based clinician-guided individual versus group treatment for social anxiety disorder. *Behav Res Ther* 84:14-26.

Titov N, Andrews G, Choi I, Schwencke G, Mahoney A (2008a): Shyness 3: randomized controlled trial of guided versus unguided Internet-based CBT for social phobia. *Aust N Z J Psychiatry* 42:1030-40.

Titov N, Andrews G, Robinson E, Schwencke G, Johnston L, Solley K, Choi I (2009): Clinician-assisted Internet-based treatment is effective for generalized anxiety disorder: randomized controlled trial. *Australian and New Zealand Journal of Psychiatry* 43:905-912.

Titov N, Andrews G, Schwencke G (2008b): Shyness 2: treating social phobia online: replication and extension. *Aust N Z J Psychiatry* 42:595-605.

Titov N, Andrews G, Schwencke G, Drobny J, Einstein D (2008c): Shyness 1: distance treatment of social phobia over the Internet. *Aust N Z J Psychiatry* 42:585-94.

Titov N, Andrews G, Schwencke G, Robinson E, Peters L, Spence J (2010): Randomized controlled trial of Internet cognitive behavioural treatment for social phobia with and without motivational enhancement strategies. *Australian and New Zealand Journal of Psychiatry* 44:938-945.

Tulbure BT, Szentagotai A, David O, Stefan S, Mansson KN, David D, Andersson G (2015): Internet-delivered cognitive-behavioral therapy for social anxiety disorder in Romania: a randomized controlled trial. *PLoS One* 10:e0123997.

Wims E, Titov N, Andrews G, Choi I (2010): Clinician-assisted Internet-based treatment is effective for panic: A randomized controlled trial. *Aust N Z J Psychiatry* 44:599-607.

**List of Excluded Studies**

Supplementary table 2: Excluded Studies. Reasons for exclusion: (1) review/comment (n=6); (2) open study/case report (n=12); (3) no primary anxiety disorder diagnosis (n=1); (4) sample of mixed diagnoses (n=4); (5) only subgroups included (n=6); (6) secondary analysis (n=8); (7) inclusion criteria not fulfilled (n=35); (8) only report of follow-up analysis (n=3)

|  |  | **Reasons for exclusion** | | | | | | | |
| --- | --- | --- | --- | --- | --- | --- | --- | --- | --- |
| **No.** | **Reference** | **1** | **2** | **3** | **4** | **5** | **6** | **7** | **8** |
|  | Alavi NAZANIN, Hirji ALYSSA: **The Efficacy of PowerPoint-based CBT Delivered Through Email: Breaking the Barriers to Treatment for Generalized Anxiety Disorder**. *Journal of Psychiatric Practice* 2020, **26**(2):89-100. |  |  |  |  |  |  | x |  |
|  | Andersson G, Carlbring P, Grimund A: **Predicting treatment outcome in internet versus face to face treatment of panic disorder**. *Comput Hum Behav* 2008, **24**(5):1790-1801. |  |  |  |  |  | x |  |  |
|  | Andersson G, Carlbring P: **Commentary on Berger, Hohl, and Caspar's (2009) Internet-based treatment for social phobia: a randomized controlled trial**. *J Clin Psychol* 2009, **65**(10):1036-1038. | x |  |  |  |  |  |  |  |
|  | Andersson G, Carlbring P, Furmark T, Grp SR: **Therapist Experience and Knowledge Acquisition in Internet-Delivered CBT for Social Anxiety Disorder: A Randomized Controlled Trial**. *Plos One* 2012, **7**(5). |  |  |  |  |  |  | x |  |
|  | Andersson G, Paxling B, Wiwe M, Vernmark K, Felix CB, Lundborg L, Furmark T, Cuijpers P, Carlbring P: **Therapeutic alliance in guided internet-delivered cognitive behavioural treatment of depression, generalized anxiety disorder and social anxiety disorder**. *Behaviour Research and Therapy* 2012, **50**(9):544-550. |  |  |  |  |  |  | x |  |
|  | Andersson E, Ruck C, Lavebratt C, Hedman E, Schalling M, Lindefors N, Eriksson E, Carlbring P, Andersson G, Furmark T: **Genetic Polymorphisms in Monoamine Systems and Outcome of Cognitive Behavior Therapy for Social Anxiety Disorder**. *Plos One* 2013, **8**(11). |  |  |  |  |  | x |  |  |
|  | Berger T, Hohl E, Caspar F: **Internet-based treatment for social phobia: A 6-month follow-up**. *Zeitschrift fur klinische Psychologie und Psychotherapie* 2010, **39**(4):217-221. |  |  |  |  |  |  |  | x |
|  | Berger T, Caspar F, Richardson R, Kneubuehler B, Sutter D, Andersson G: **Internet-based treatment of social phobia: A randomized controlled trial comparing unguided with two types of guided self-help**. *Behaviour Research and Therapy* 2011, **49**(3):158-169. |  |  |  |  |  |  | x |  |
|  | Bergström J, Andersson G, Karlsson A, Andréewitch S, Rück C, Carlbring P, Lindefors N: **An open study of the effectiveness of Internet treatment for panic disorder delivered in a psychiatric setting**. *Nord J Psychiatry* 2009, **63**(1):44-50. |  | x |  |  |  |  |  |  |
|  | Boettcher J, Berger T, Renneberg B: **Does a pre-treatment diagnostic interview affect the outcome of internet-based self-help for social anxiety disorder? a randomized controlled trial**. *Behav Cogn Psychother* 2012, **40**(5):513-528. |  |  |  |  |  |  | x |  |
|  | Boettcher J, Carlbring P, Renneberg B, Berger T: **Internet-Based Interventions for Social Anxiety Disorder - an Overview**. *Verhaltenstherapie* 2013, **23**(3):160-168. | x |  |  |  |  |  |  |  |
|  | Bruinsma A, Kampman M, Exterkate CC, Hendriks GJ: **[An exploratory study of 'blended' cognitive behavioural therapy (CBT) for patients with a panic disorder: results and patients' experiences]**. *Tijdschrift voor psychiatrie* 2016, **58**(5):361-370. |  |  |  |  |  |  | x |  |
|  | Carlbring P, Brunt S, Bohman S, Austin D, Richards J, Ost L-G, Andersson G: **Internet vs. paper and pencil administration of questionnaires commonly used in panic/agoraphobia research**. *Comput Hum Behav* 2007, **23**(3):1421-1434. |  |  |  |  |  |  | x |  |
|  | Carlbring P, Apelstrand M, Sehlin H, Amir N, Rousseau A, Hofmann SG, Andersson G: **Internet-delivered attention bias modification training in individuals with social anxiety disorder - a double blind randomized controlled trial**. *BMC psychiatry* 2012, **12**. |  |  |  |  |  |  | x |  |
|  | Cervenka S, Hedman E, Ikoma Y, Djurfeldt DR, Rück C, Halldin C, Lindefors N: **Changes in dopamine D2-receptor binding are associated to symptom reduction after psychotherapy in social anxiety disorder**. *Transl Psychiatry* 2012, **2**(5):e120. |  |  |  |  |  |  | x |  |
|  | Christensen H, Batterham P, Mackinnon A, Griffiths KM, Hehir KK, Kenardy J, Gosling J, Bennett K: **Prevention of Generalized Anxiety Disorder Using a Web Intervention, iChill: Randomized Controlled Trial**. *Journal of Medical Internet Research* 2014, **16**(9). |  |  |  |  |  |  | x |  |
|  | Christoforou M, Fonseca JAS, Tsakanikos E: **Two Novel Cognitive Behavioral Therapy-Based Mobile Apps for Agoraphobia: Randomized Controlled Trial**. *Journal of Medical Internet Research* 2017, **19**(11). |  |  |  |  |  |  | x |  |
|  | Draper M, Rees CS, Nathan PR: **Internet-Based Self-Management of Generalised Anxiety Disorder: A Preliminary Study**. *Behav Change* 2008, **25**(4):229-244. |  | x |  |  |  |  |  |  |
|  | Duval ER, Joshi SA, Block SR, Abelson JL, Liberzon I: **Insula activation is modulated by attention shifting in social anxiety disorder**. *J Anx Dis* 2018, **56**:56-62. |  | x |  |  |  |  |  |  |
|  | Ebenfeld L, Stegemann SK, Lehr D, Ebert DD, Funk B, Riper H, Berking M: **A mobile application for panic disorder and agoraphobia: Insights from a multi-methods feasibility study**. *Internet Interventions-the Application of Information Technology in Mental and Behavioural Health* 2020, **19**. |  | x |  |  |  |  |  |  |
|  | El Alaoui S, Hedman E, Ljótsson B, Bergström J, Andersson E, Rück C, Andersson G, Lindefors N: **Predictors and moderators of internet- and group-based cognitive behaviour therapy for panic disorder**. *PLoS One* 2013, **8**(11):e79024. |  |  |  |  |  | x |  |  |
|  | El Alaoui S, Hedman-Lagerlöf E, Ljótsson B, Lindefors N: **Does internet-based cognitive behaviour therapy reduce healthcare costs and resource use in treatment of social anxiety disorder? A cost-minimisation analysis conducted alongside a randomised controlled trial**. *Bmj Open* 2017, **7**(9):e017053. |  |  |  |  |  | x |  |  |
|  | Fogliati VJ, Terides MD, Gandy M, Staples LG, Johnston L, Karin E, Rapee RM, Titov N, Dear BF: **Psychometric properties of the mini-social phobia inventory (Mini-SPIN) in a large online treatment-seeking sample**. *Cognitive behaviour therapy* 2016, **45**(3):236-257. |  |  |  |  |  |  | x |  |
|  | Gershkovich M, Herbert JD, Forman EM, Glassman L: **Guided Internet-Based Self-Help Intervention for Social Anxiety Disorder With Videoconferenced Therapist Support**. *Cogn Behav Pract* 2016, **23**(2):239-255. |  | x |  |  |  |  |  |  |
|  | Hedman E, Furmark T, Carlbring P, Ljotsson B, Ruck C, Lindefors N, Andersson G: **A 5-Year Follow-up of Internet-Based Cognitive Behavior Therapy for Social Anxiety Disorder**. *Journal of Medical Internet Research* 2011, **13**(2). |  |  |  |  |  |  |  | x |
|  | Hedman E, Andersson E, Ljótsson B, Andersson G, Rück C, Lindefors N: **Cost-effectiveness of Internet-based cognitive behavior therapy vs. cognitive behavioral group therapy for social anxiety disorder: results from a randomized controlled trial**. *Behaviour Research and Therapy* 2011, **49**(11):729-736. |  |  |  |  |  | x |  |  |
|  | Hedman E, Andersson E, Ljótsson B, Andersson G, Andersson E, Schalling M, Lindefors N, Rück C: **Clinical and genetic outcome determinants of Internet- and group-based cognitive behavior therapy for social anxiety disorder**. *Acta Psychiatr Scand* 2012, **126**(2):126-136. |  |  |  |  |  | x |  |  |
|  | Hedman E, Ljótsson B, Rück C, Bergström J, Andersson G, Kaldo V, Jansson L, Andersson E, Andersson E, Blom K *et al*: **Effectiveness of internet-based cognitive behaviour therapy for panic disorder in routine psychiatric care**. *Acta Psychiatr Scand* 2013, **128**(6):457-467. |  |  |  |  |  | x |  |  |
|  | Hedman E, El Alaoui S, Lindefors N, Andersson E, Rück C, Ghaderi A, Kaldo V, Lekander M, Andersson G, Ljótsson B: **Clinical effectiveness and cost-effectiveness of Internet- vs. group-based cognitive behavior therapy for social anxiety disorder: 4-year follow-up of a randomized trial**. *Behaviour Research and Therapy* 2014, **59**:20-29. |  |  |  |  |  |  |  | x |
|  | Hobbs MJ, Mahoney AEJ, Andrews G: **Integrating iCBT for generalized anxiety disorder into routine clinical care: Treatment effects across the adult lifespan**. *J Anx Dis* 2017, **51**:47-54. |  |  |  |  |  |  | x |  |
|  | Ivanova E, Lindner P, Ly KH, Dahlin M, Vernmark K, Andersson G, Carlbring P: **Guided and unguided Acceptance and Commitment Therapy for social anxiety disorder and/or panic disorder provided via the Internet and a smartphone application: A randomized controlled trial**. *Journal of Anxiety Disorders* 2016, **44**:27-35. |  |  |  | x |  |  |  |  |
|  | Jager J, Emmelkamp PMG, Lange A: **Treatment of panic disorder by Internet: A case study**. *Verhaltenstherapie* 2004, **14**(3):200-205. |  | x |  |  |  |  |  |  |
|  | Kaehlke F, Berger T, Schulz A, Baumeister H, Berking M, Auerbach RP, Bruffaerts R, Cuijpers P, Kessler RC, Ebert DD: **Efficacy of an unguided internet-based self-help intervention for social anxiety disorder in university students: A randomized controlled trial**. *International Journal of Methods in Psychiatric Research* 2019, **28**(2). |  |  |  |  | x |  |  |  |
|  | Kanuri N, Newman MG, Ruzek JI, Kuhn E, Manjula M, Jones M, Thomas N, Abbott J-AM, Sharma S, Taylor CB: **The Feasibility, Acceptability, and Efficacy of Delivering Internet-Based Self-Help and Guided Self-Help Interventions for Generalized Anxiety Disorder to Indian University Students: Design of a Randomized Controlled Trial**. *JMIR research protocols* 2015, **4**(4):e136-e136. |  |  |  |  | x |  |  |  |
|  | Koszycki D, Taljaard M, Segal Z, Bradwejn J: **A randomized trial of sertraline, self-administered cognitive behavior therapy, and their combination for panic disorder**. *Psychological Medicine* 2011, **41**(2):373-383. |  |  |  |  |  |  | x |  |
|  | Kuckertz JM, Gildebrant E, Liliequist B, Karlström P, Väppling C, Bodlund O, Stenlund T, Hofmann SG, Andersson G, Amir N *et al*: **Moderation and mediation of the effect of attention training in social anxiety disorder**. *Behaviour Research and Therapy* 2014, **53**:30-40. |  |  |  |  |  |  | x |  |
|  | Lampe LA: **Social anxiety disorder: recent developments in psychological approaches to conceptualization and treatment**. *Australian and New Zealand Journal of Psychiatry* 2009, **43**(10):887-898. | x |  |  |  |  |  |  |  |
|  | Lindegaard T, Hesslow T, Nilsson M, Johansson R, Carlbring P, Lilliengren P, Andersson G: **Internet-based psychodynamic therapy vs cognitive behavioural therapy for social anxiety disorder: A preference study**. *Internet Interventions-the Application of Information Technology in Mental and Behavioural Health* 2020, **20**. |  |  |  |  |  |  | x |  |
|  | Lindner P, Carlbring P, Flodman E, Hebert A, Poysti S, Hagkvist F, Johansson R, Westin VZ, Berger T, Andersson G: **Does cognitive flexibility predict treatment gains in Internet-delivered psychological treatment of social anxiety disorder, depression, or tinnitus?** *PeerJ* 2016, **4**. |  |  |  |  |  |  | x |  |
|  | Lonsdorf TB, Rück C, Bergström J, Andersson G, Ohman A, Lindefors N, Schalling M: **The COMTval158met polymorphism is associated with symptom relief during exposure-based cognitive-behavioral treatment in panic disorder**. *BMC psychiatry* 2010, **10**:99. |  |  |  |  |  |  | x |  |
|  | Lorian CN, Titov N, Grisham JR: **Changes in risk-taking over the course of an internet-delivered cognitive behavioral therapy treatment for generalized anxiety disorder**. *Journal of Anxiety Disorders* 2012, **26**(1):140-149. |  |  |  |  |  |  | x |  |
|  | Mahoney AEJ, Newby JM, Hobbs MJ, Williams AD, Andrews G: **Reducing behavioral avoidance with internet-delivered cognitive behavior therapy for generalized anxiety disorder**. *Internet Interventions-the Application of Information Technology in Mental and Behavioural Health* 2019, **15**:105-109. |  | x |  |  |  |  |  |  |
|  | Mansson KNT, Carlbring P, Frick A, Engman J, Olsson C-J, Bodlund O, Furmark T, Andersson G: **Altered neural correlates of affective processing after internet-delivered cognitive behavior therapy for social anxiety disorder**. *Psychiatry Res Neuroimaging* 2013, **214**(3):229-237. |  |  |  |  |  |  | x |  |
|  | Månsson KNT, Salami A, Carlbring P, Boraxbekk CJ, Andersson G, Furmark T: **Structural but not functional neuroplasticity one year after effective cognitive behaviour therapy for social anxiety disorder**. *Behav Brain Res* 2017, **318**:45-51. |  |  |  |  |  |  | x |  |
|  | Mathiasen K, Riper H, Ehlers LH, Valentin JB, Rosenberg NK: **Internet-based CBT for social phobia and panic disorder in a specialised anxiety clinic in routine care: Results of a pilot randomised controlled trial**. *Internet interventions* 2016, **4**:92-98. |  |  |  | x |  |  |  |  |
|  | Matsumoto K, Sutoh C, Asano K, Seki Y, Urao Y, Yokoo M, Takanashi R, Yoshida T, Tanaka M, Noguchi R *et al*: **Internet-Based Cognitive Behavioral Therapy With Real-Time Therapist Support via Videoconference for Patients With Obsessive-Compulsive Disorder, Panic Disorder, and Social Anxiety Disorder: Pilot Single-Arm Trial**. *Journal of Medical Internet Research* 2018, **20**(12). |  |  |  | x |  |  |  |  |
|  | Miller CB, Gu J, Henry AL, Davis ML, Espie CA, Stott R, Heinz AJ, Bentley KH, Goodwin GM, Gorman BS *et al*: **Feasibility and efficacy of a digital CBT intervention for symptoms of Generalized Anxiety Disorder: A randomized multiple-baseline study**. *J Behav Ther Exp Psychiatry* 2021, **70**:101609. |  | x |  |  |  |  |  |  |
|  | Neubauer K, von Auer M, Murray E, Petermann F, Helbig-Lang S, Gerlach AL: **Internet-delivered attention modification training as a treatment for social phobia: a randomized controlled trial**. *Behaviour Research and Therapy* 2013, **51**(2):87-97. |  |  |  |  |  |  | x |  |
|  | Nordgreen T, Standal B, Mannes H, Haug T, Sivertsen B, Carlbring P, Andersson G, Heiervang E, Havik OE: **Guided self-help via internet for panic disorder: Dissemination across countries**. *Comput Hum Behav* 2010, **26**(4):592-596. |  | x |  |  |  |  |  |  |
|  | Nordgreen T, Havik OE, Ost LG, Furmark T, Carlbring P, Andersson G: **Outcome predictors in guided and unguided self-help for social anxiety disorder**. *Behaviour Research and Therapy* 2012, **50**(1):13-21. |  |  |  |  |  |  | x |  |
|  | Nordgreen T, Haug T, Ost L-G, Andersson G, Carlbring P, Kvale G, Tangen T, Heiervang E, Havik OE: **Stepped Care Versus Direct Face-to-Face Cognitive Behavior Therapy for Social Anxiety Disorder and Panic Disorder: A Randomized Effectiveness Trial**. *Behavior Therapy* 2016, **47**(2):166-183. |  |  |  | x |  |  |  |  |
|  | Nordgreen T, Gjestad R, Andersson G, Carlbring P, Havik OE: **The implementation of guided Internet-based cognitive behaviour therapy for panic disorder in a routine-care setting: effectiveness and implementation efforts**. *Cognitive behaviour therapy* 2018, **47**(1):62-75. |  |  |  |  |  |  | x |  |
|  | Nordh M, Vigerland S, Ost L-G, Ljotsson B, Mataix-Cols D, Serlachius E, Hogstrom J: **Therapist-guided internet-delivered cognitive-behavioural therapy supplemented with group exposure sessions for adolescents with social anxiety disorder: a feasibility trial**. *Bmj Open* 2017, **7**(12). |  |  |  |  | x |  |  |  |
|  | Nordin S, Carlbring P, Cuijpers P, Andersson G: **Expanding the Limits of Bibliotherapy for Panic Disorder: Randomized Trial of Self-Help Without Support but With a Clear Deadline**. *Behavior Therapy* 2010, **41**(3):267-276. |  |  |  |  |  |  | x |  |
|  | Paxling B, Lundgren S, Norman A, Almlöv J, Carlbring P, Cuijpers P, Andersson G: **Therapist behaviours in internet-delivered cognitive behaviour therapy: analyses of e-mail correspondence in the treatment of generalized anxiety disorder**. *Behav Cogn Psychother* 2013, **41**(3):280-289. |  |  |  |  |  | x |  |  |
|  | Pelissolo A, Abou Kassm S, Delhay L: **Therapeutic strategies for social anxiety disorder: where are we now?** *Expert review of neurotherapeutics* 2019, **19**(12):1179-1189. | x |  |  |  |  |  |  |  |
|  | Probst T, Berger T, Meyer B, Spaeth C, Schroeder J, Hohagen F, Moritz S, Klein JP: **Social Phobia Moderates the Outcome in the EVIDENT Study: A Randomized Controlled Trial on an Internet-Based Psychological Intervention for Mild to Moderate Depressive Symptoms**. *Journal of consulting and clinical psychology* 2020, **88**(1):82-89. |  |  | x |  |  |  |  |  |
|  | Schneider AJ, Mataix-Cols D, Marks IM, Bachofen M: **Internet-guided self-help with or without exposure therapy for phobic and panic disorders - A randomised controlled trial**. *Psychotherapy and Psychosomatics* 2005, **74**(3):154-164. |  |  |  |  |  |  | x |  |
|  | Shandley K, Austin DW, Klein B, Pier C, Schattner P, Pierce D, Wade V: **Therapist-Assisted, Internet-Based Treatment for Panic Disorder: Can General Practitioners Achieve Comparable Patient Outcomes to Psychologists?** *Journal of Medical Internet Research* 2008, **10**(2). |  |  |  |  |  |  | x |  |
|  | Spence SH, Donovan CL, March S, Kenardy JA, Hearn CS: **Generic versus disorder specific cognitive behavior therapy for social anxiety disorder in youth: A randomized controlled trial using internet delivery**. *Behaviour Research and Therapy* 2017, **90**:41-57. |  |  |  |  | x |  |  |  |
|  | Stangier U: **New Developments in Cognitive-Behavioral Therapy for Social Anxiety Disorder**. *Current Psychiatry Reports* 2016, **18**(3). | x |  |  |  |  |  |  |  |
|  | Stech EP, Grierson AB, Chen AZ, Sharrock MJ, Mahoney AEJ, Newby JM: **Intensive one-week internet-delivered cognitive behavioral therapy for panic disorder and agoraphobia: A pilot study**. *Internet Interv* 2020, **20**:100315. |  | x |  |  |  |  |  |  |
|  | Stolz T, Schulz A, Krieger T, Vincent A, Urech A, Moser C, Westermann S, Berger T: **A mobile app for social anxiety disorder: A three-arm randomized controlled trial comparing mobile and PC-based guided self-help interventions**. *Journal of consulting and clinical psychology* 2018, **86**(6):493-504. |  |  |  |  |  |  | x |  |
|  | Stott R, Wild J, Grey N, Liness S, Warnock-Parkes E, Commins S, Readings J, Bremner G, Woodward E, Ehlers A *et al*: **Internet-delivered cognitive therapy for social anxiety disorder: a development pilot series**. *Behav Cogn Psychother* 2013, **41**(4):383-397. |  | x |  |  |  |  |  |  |
|  | Thew GR, Powell CL, Kwok AP, Lissillour Chan MH, Wild J, Warnock-Parkes E, Leung PW, Clark DM: **Internet-Based Cognitive Therapy for Social Anxiety Disorder in Hong Kong: Therapist Training and Dissemination Case Series**. *JMIR Form Res* 2019, **3**(2):e13446. |  |  |  |  |  |  | x |  |
|  | Tillfors M, Carlbring P, Furmark T, Lewenhaupt S, Spak M, Eriksson A, Westling BE, Andersson G: **Treating university students with social phobia and public speaking fears: Internet delivered self-help with or without live group exposure sessions**. *Depress Anxiety* 2008, **25**(8):708-717. |  |  |  |  | x |  |  |  |
|  | Tillfors M, Andersson G, Ekselius L, Furmark T, Lewenhaupt S, Karlsson A, Carlbring P: **A randomized trial of Internet-delivered treatment for social anxiety disorder in high school students**. *Cognitive behaviour therapy* 2011, **40**(2):147-157. |  |  |  |  | x |  |  |  |
|  | Tillfors M, Furmark T, Carlbring P, Andersson G: **Risk profiles for poor treatment response to internet-delivered CBT in people with social anxiety disorder**. *Journal of Anxiety Disorders* 2015, **33**:103-109. |  |  |  |  |  |  | x |  |
|  | Titov N, Andrews G, Schwencke G, Solley K, Johnston L, Robinson E: **An RCT comparing effect of two types of support on severity of symptoms for people completing Internet-based cognitive behaviour therapy for social phobia**. *Australian and New Zealand Journal of Psychiatry* 2009, **43**(10):920-926. |  |  |  |  |  |  | x |  |
|  | Titov N, Andrews G, Choi I, Schwencke G, Johnston L: **Randomized controlled trial of web-based treatment of social phobia without clinician guidance**. *Australian and New Zealand Journal of Psychiatry* 2009, **43**(10):913-919. |  |  |  |  |  |  | x |  |
|  | Titov N, Gibson M, Andrews G, McEvoy P: **Internet treatment for social phobia reduces comorbidity**. *Aust N Z J Psychiatry* 2009, **43**(8):754-759. |  |  |  |  |  |  | x |  |
|  | Van Singer M, Chatton A, Khazaal Y: **Quality of smartphone apps related to panic disorder**. *Frontiers in psychiatry* 2015, **6**. | x |  |  |  |  |  |  |  |
|  | Walderhaug EP, Gjestad R, Egeland J, Havik OE, Nordgreen T: **Relationships between depressive symptoms and panic disorder symptoms during guided internet-delivered cognitive behavior therapy for panic disorder**. *Nord J Psychiat* 2019, **73**(7):417-424. |  |  |  |  |  |  | x |  |
|  | Wang H, Zhao Q, Mu W, Rodriguez M, Qian M, Berger T: **The Effect of Shame on Patients With Social Anxiety Disorder in Internet-Based Cognitive Behavioral Therapy: Randomized Controlled Trial**. *JMIR Ment Health* 2020, **7**(7):e15797. |  |  |  |  |  |  | x |  |
|  | Yuen EK, Herbert JD, Forman EM, Goetter EM, Juarascio AS, Rabin S, Goodwin C, Bouchard S: **Acceptance based behavior therapy for social anxiety disorder through videoconferencing**. *J Anx Dis* 2013, **27**(4):389-397. |  | x |  |  |  |  |  |  |
| N |  | 6 | 12 | 1 | 4 | 6 | 8 | 35 | 3 |
